# Heart Rate Variability, HIV and the Risk of Cardiovascular Diseases in Rural South Africa

**DOI:** 10.5334/gh.532

**Published:** 2020-02-12

**Authors:** Noortje G. Godijk, Alinda G. Vos, Vita W. Jongen, Robert Moraba, Hugo Tempelman, Diederick E. Grobbee, Roel A. Coutinho, Walter Devillé, Kerstin Klipstein-Grobusch

**Affiliations:** 1 Julius Global Health, Julius Center for Health Sciences and Primary Care, University Medical Center Utrecht, Utrecht University, Utrecht, NL; 2 Wits Reproductive Health and HIV Institute, Faculty of Health Sciences, University of the Witwatersrand, Johannesburg, ZA; 3 Ndlovu Care Group, Groblersdal, ZA; 4 Division of Epidemiology and Biostatistics, School of Public Health, Faculty of Health Sciences, University of the Witwatersrand, Johannesburg, ZA

**Keywords:** heart rate variability, HIV, antiretroviral treatment, sub-Saharan Africa, cardiovascular disease

## Abstract

**Background:**

Antiretroviral therapy (ART) transformed human immunodeficiency virus (HIV) infection into a chronic disease. Possible HIV-associated complications have emerged including cardiovascular diseases (CVD).

**Objectives:**

This study aims to determine the heart rate variability (HRV) distribution and association between HRV and HIV treated with ART in a rural African population.

**Methods:**

This cross-sectional study included 325 participants of the Ndlovu Cohort Study, South Africa. HRV was measured using a standardized five-minute resting ECG and assessed by the standard deviation of normal RR intervals (SDNN), root of mean squares of successive RR differences (RMSSD), percentage of RR intervals greater than 50 milliseconds different from its predecessor (pNN50), total-, low- and high-frequency power. CVD risk factors were assessed using measurements (blood pressure, anthropometry, cholesterol) and questionnaires (e.g. socio-demographics, alcohol, smoking, physical activity, age, diabetes). We used a Wilcoxon rank test to assess differences in medians between HIV-infected and HIV-uninfected participants and multivariable linear regression to investigate associations between HRV and HIV treated with ART.

**Conclusions:**

Of the participants, 196 (61.4%) were HIV-infected treated with ART and 123 (38.6%) were HIV-uninfected. HIV-infected consumed less alcohol, 52% versus 35%, smoked less, were less physically active, more often attained lower education, 26% versus 14%, and had lower systolic blood pressure, 134 mmHg versus 140 mmHg, compared to HIV-uninfected. Medians of all HRV parameters were lower for HIV-infected participants. The model fully adjusted for CVD risk factors showed a significant inverse association between HIV treated with ART and log RMSSD (–0.16) and log pnn50 (–0.61). Although HIV-infected participants treated with ART presented with less CVD risk factors they had a lower HRV indicating an increased risk of CVD.

**Highlights:**

– African HIV-infected participants on ART had less conventional CVD risk factors than HIV-uninfected.
– However, HIV-infected participants had lower HRV than HIV-uninfected participants.
– Lower HRV of the HIV-infected participants indicates that they are at a higher risk for CVD.

## 1. Introduction

In 2018, an estimated 62% of all people with human immunodeficiency virus (HIV) globally received antiretroviral treatment (ART) [1]. Long-term treatment with ART increases the life expectancy of HIV-patients and has transformed HIV into a chronic disease [2]. With the ageing of the HIV-infected population, possible HIV-associated complications have emerged, most notably cardiovascular diseases (CVD) [3,4]. Apart from conventional CVD risk factors, HIV-infected individuals may be at an increased risk of CVD due to HIV-infection, possibly related to immune activation while certain antivirals may also play a role [4,5]. A recent meta-analysis reported that people living with HIV have a twofold increased risk of cardiovascular disease [6].

Although approximately 25.5 million HIV-infected individuals – nearly 70% of all people infected with HIV – live in sub-Saharan Africa (SSA), research on the association between HIV and CVD in this area is scarce. The differences in lifestyle, socio-economic factors and HIV subtype preclude generalization of data from Western countries to the SSA regions.

One way of estimating CVD risk is by using surrogate markers [7]. Heart rate variability (HRV) has been shown to be an independent predictor of CVD [8,9,10]. High HRV is a sign of good adaptation and efficient autonomic mechanisms. Conversely, low HRV is often an indicator of inadequate adaptation of the autonomic nervous system (ANS) [11]. Autonomic dysfunction was commonly detected in HIV and AIDS patients prior to the advent of ART, suggesting an autonomic neuropathological effect of HIV [10]. However, the effect of HIV on HRV in the current era of widespread ART availability is more ambiguous [10,12]. Research on a small group of HIV-infected subjects treated with ART demonstrated a decrease in all parameters using 24-hour HRV, although another study did not find a difference in parasympathetic activity but only in total HRV [13]. Research on the distribution of HRV in HIV-infected and HIV-uninfected populations in SSA which takes into account classical CVD risk factors and ART allows to further assess the link between HIV, ART, CVD risk and autonomic function [14].

The aim of this study is twofold. First, we assessed differences between CVD risk factors of HIV-infected treated with ART compared to HIV-uninfected subjects. Second, we investigated the distribution of HRV and the difference between HRV of HIV-infected treated with ART compared to HIV-uninfected subjects while taking CVD risk factors into account.

## 2. Methods

### 2.1 Study design

This cross-sectional study was embedded in the Ndlovu Cohort study (NCS) [15].

### 2.2 Recruitment of participants

The NCS is a prospective study in the Moutse area, Limpopo Province, South Africa and aims to provide a comprehensive understanding of the interaction between HIV and CVD in the black SSA population. From November 2014 until August 2017 this study recruited 1,040 HIV-uninfected and 887 HIV-infected participants through community campaigns, at local events and shopping centers as well as at the Ndlovu Medical Center (NMC) HIV clinic. The NMC was a large rural HIV treatment facility, contracted by the South African Department of Health, providing free-of-charge HIV treatment and follow-up to approximately 3,700 HIV-infected patients. Criteria for eligibility were age 18 years and older, being able to provide written informed consent and be committed to long-term follow-up. Routine physical examination was used to assess CVD risk factors. Full study procedures of the NCS have been described previously [15].

All the participants of the NCS who came to the research site for a follow-up or baseline visit between August 2017 to December 2017 were approached for additional HRV assessment. Informed consent was obtained from each patient. We excluded HIV-infected not treated with ART, pregnant women and individuals unable to undergo the study procedures for any reason. The study conforms to the ethical guidelines of the 1975 Declaration of Helsinki as reflected by the ethics approval obtained from the Review Ethical Committee of the University of Pretoria (ref. number 227_2017).

### 2.3 Patient and public involvement

The NCS is well known in the community, regular community events are organized and a community liaison officer maintains the relationship with the community. However, patients and the public were not involved in the design, conduct and reporting of this research.

### 2.4 Heart rate variability

HRV was measured in laying position with the upper body at a slight upward angle during five minutes using a 12-lead computer-based ECG Sampling box (SE-1515 DP12, EDAN, 4204 Jutland Dr Suite B, San Diego, CA 92117, United States) and complementary software. All measurements were performed according to standardized procedures by two trained investigators who were unaware of the subjects’ HIV-status at the time the measurements were taken.

Time and frequency domain parameters were measured. Time-domain parameters address the magnitude of variability and provide information about the vagal (parasympathetic) modulation of the heart, with higher variability generally reflecting greater parasympathetic modulation [14]. Time-domain measures include the standard deviation of the normal RR intervals (SDNN), the root of the mean squares of successive RR differences (RMSSD) and the percentage of RR intervals greater than 50 milliseconds different from its predecessor (pNN50). Overall HRV is reflected by SDNN and RMSSD measures, with SDNN being the most representative parameter of HRV, whereas pNN50 measures HRV’s short-term components. SDNN is the most representative since it is calculated using variance, which is mathematically equal to the total power of spectral analysis, and therefore SDNN reflects all the cyclic components responsible for variability in the period during which HRV is recorded [14,16].

Frequency parameters use power spectral analysis of the beat-to-beat variations of the heart rate (R–R interval) [17]. This method divides total variance (‘power’) of a continuous series of heartbeats into frequency components [16,17]. Of the frequency parameters, three spectral components were measured: low-frequency (LF), high-frequency (HF) and total-frequency (TF) power. The influence of LF power on the autonomic nervous system is controversial, some consider LF power (LF; 0.04 to 0.15 Hertz) to represent sympathetic activity [18], although others claim it represents parasympathetic and sympathetic activity [19]. HF power (HF; 0.15 to 0.40 Hertz) reflects sympathetic activity [14,16]. TF power reflects total autonomic activity [16].

### 2.5 Other measurements

Information was collected on date of birth, sex, smoking status, alcohol use, chronic medication use and physical activity using standardized questionnaires, namely the WHO’s STEPS Instrument [20]. Physical activity was categorized as inactive and active according to the WHO definition [21]. Validated questionnaires, the National Income Dynamics Study Wave 3 for Adults, were used for information on education, employment, income and household support [22]. Income was classified as earning ≤600 South African rand (R), R600–3100, R3100–11000 and ≥R11000 per month. Education was classified as low (non or primary), middle (secondary or matric) and high (college or university). Information was collected on height, weight and blood pressure (BP). BP was measured in a seated position after a fifteen-minute rest. BP was taken at both wrists and repeated at the wrist with the highest values. The average of the second and third reading was used for the analysis. Fasting glucose, triglycerides, total, HDL and LDL cholesterol were measured by Toga labs, South Africa. In HIV-uninfected subjects, HIV status was measured according to the NCS protocol [15].

### 2.6 Statistical analysis

Due to technical errors during the HRV measurement 31 participants were excluded. The sample size was calculated a priori based on the difference between the mean SDNN of the HIV-infected treated with ART and uninfected. The difference was estimated to be 6, with a standard deviation of 18 [12]. The minimal required sample size to reach a power of 80% and a significance of 95% was estimated to be 143 participants in each group.

Missing data for medication use, BMI, systolic and diastolic BP were less than one percentage. A t-test and a Wilcoxon rank test were used to test for differences in continuous variables. Chi-square test was used to test for a difference in the categorical variables: smoking, alcohol use, income and educational level. The difference in the distributions of median HRV between HIV-infected treated with ART and HIV-uninfected participants was assessed using the Wilcoxon rank test.

HRV parameters were log-transformed to reach a normal distribution. Linear regression analysis investigated the association of HIV and HRV parameters. In model 1 the association between HIV and HRV was investigated, while adjusting for age and sex. In model 2 the association was further adjusted for all CVD risk factors with a p-value < 0.2 in the univariable analysis. Model 3 was additionally adjusted for socio-economic variables. Data were analysed using R version 3.4.0. [23] and a two-sided p-value of 0.05 was considered statistically significant.

## 3. Results

The study population comprised 319 participants, of whom 196 (61.4%) were HIV-infected and all were treated with ART (Table 1). The HIV-infected participants were more often women, were on average about five years older and were less educated than the HIV-uninfected participants. HIV-infected participants had lower systolic blood pressure, higher glucose, used significantly less alcohol, smoked less and were less physically active than HIV-uninfected participants.

**Table 1:** Baseline characteristics and descriptive statistics of study participants.

Variable		HIV– (n = 123)		HIV+ (n = 196)		p-value

Characteristics (n, %)						
Age (mean, SD)		38.9	(12.8)	43.8	(9.3)	<0.001
Sex	Woman	43	(35%)	108	(55%)	<0.001
Education	Low	17	(14%)	51	(26%)	<0.001
	Middle	85	(69%)	137	(70%)	
	High	21	(17%)	8	(4%)	
Income	≤R600	90	(73%)	121	(63%)	<0.04
	R600–3100	11	(9%)	19	(10%)	
	R3100–11000	15	(12%)	47	(24%)	
	≥R11000	7	(6%)	6	(3%)	
Smoking	Never	63	(51%)	128	(65%)	0.04
	Past	19	(15%)	18	(9%)	
	Current	41	(33%)	50	(26%)	
Alcohol	Never	43	(35%)	102	(52%)	<0.001
	Past	20	(16%)	32	(16%)	
	Current	60	(49%)	62	(32%)	
Physical Activity	Active	89	(72%)	103	(52%)	<0.001
Diabetes		4	(3%)	7	(4%)	1
Medication for	Diabetes	2	(2%)	2	(1%)	0.63
	High BP	5	(4%)	12	(6%)	0.57
ART first line (TDF+ FTC+ EFV)		0	0%	182	(93%)	
ART second line (AZT+ 3TC+ LPV/r or AZT +TDF+3TC+LPV/r)		0	0%	14	(7%)	
Physical measurements						
BMI kg/m^2^ (median, IQR)		22.7	(20.8–27.7)	23.8	(20.5–27.7)	0.72
Systolic BP (mmHg; mean, SD)		140	(16)	134	(16)	0.01
Diastolic BP (mmHg; mean, SD)		98	(16)	97	(15)	0.71
Glucose (median, IQR)		4.5	(4.1–4.9)	4.7	(4.4–5.2)	0.01
Total cholesterol (median, IQR)		4.0	(3.5–4.9)	4.0	(3.6–4.9)	0.89
HDL cholesterol (median, IQR)		1.4	(1.2–1.6)	1.5	(1.3–1.7)	0.6
LDL cholesterol (median, IQR)		2.2	(1.7–2.9)	2.00	(1.7–2.7)	0.23
Triglycerides (median, IQR)		0.9	(0.7–1.3)	1	(0.7–1.5)	0.12

SD; standard deviation, IQR; inter quartile range, ART; antiretroviral therapy, TDF; tenofovir, FTC; emtricitabine or lamivudine, EFV; efavirenz, AZT; zidovudine, 3TC; lamivudine, LPV/r; lopinavir/ritonavir, BP; blood pressure, HDL; high-density lipoprotein, LDL; low-density lipoprotein.

**Figure 1:**
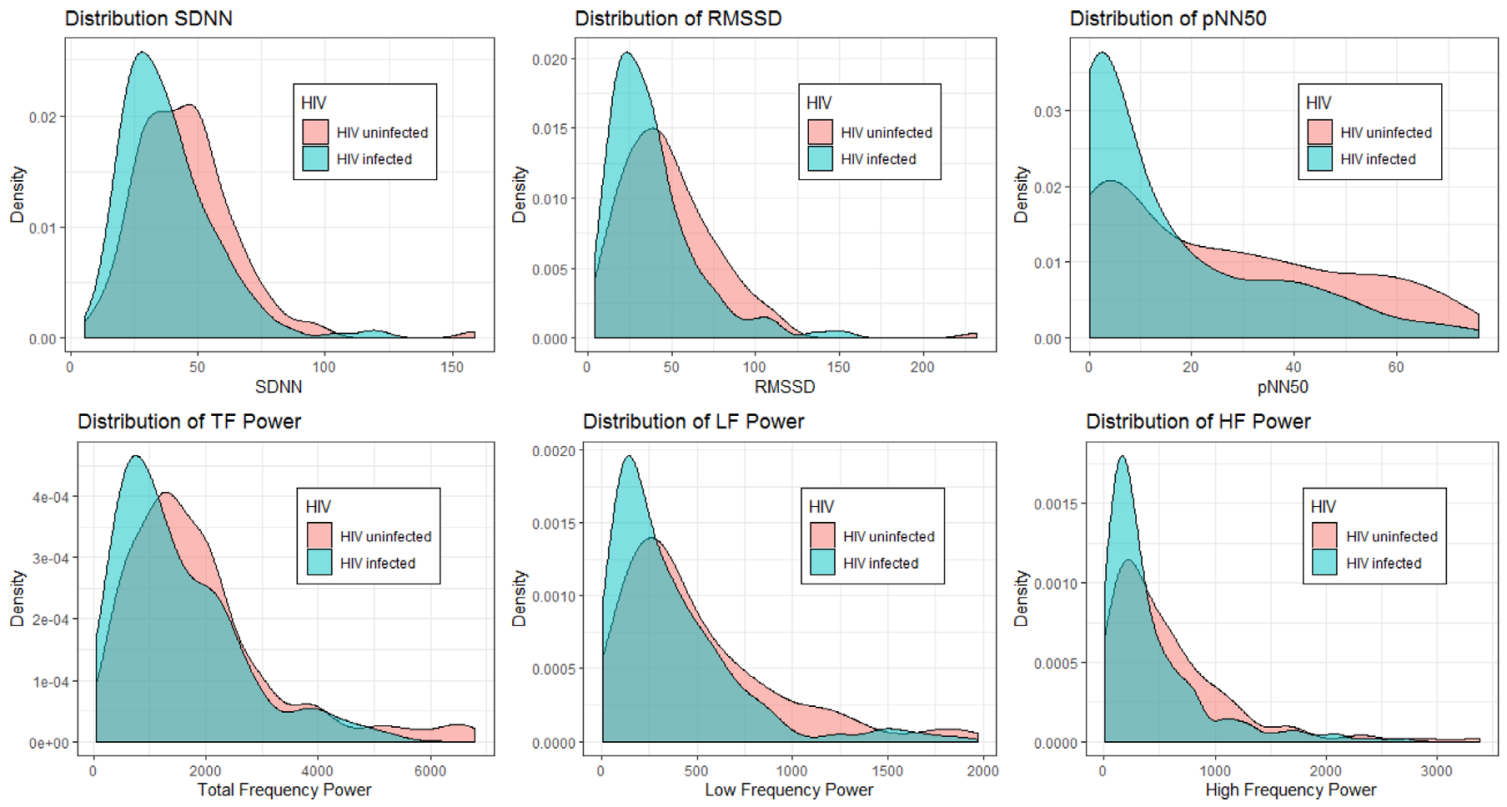
Distribution of heart rate variability. SDNN; standard deviation of the normal RR intervals, RMSSD; the root of the mean squares of successive RR differences, pNN50; the percentage of RR intervals greater than fifty milliseconds different from its predecessor, TF; total-frequency, LF; low-frequency and HF; high-frequency power.

The medians of both time and frequency domain parameters were significantly lower for HIV-infected participants compared to the HIV-uninfected participants (Table 2). The distribution of all HRV parameters was skewed to the right (Figure 1).

**Table 2:** Distribution of heart rate variability.

Variable	HIV status	Mean	Median (IQR)	p-value	Min.	Max.	Spread

TF Power	HIV–	1874.1	1478.5 (1025.5–2278.2)	<0.01	126.9	6792.5	4918.4
	HIV+	1495.4	1191.7 (647.7–2119.2)		53.05	5264.6	5211.6
LF Power	HIV–	504.1	356.2 (203.9–673.8)	<0.001	43.2	1968.9	1925.7
	HIV+	364.2	258.5 (127.8–496.1)		4.9	1885.7	1880.8
HF Power	HIV–	579.4	424.5 (192.6–765.3)	<0.01	12.9	3382.0	3369.0
	HIV+	426.3	265.8 (133.1–536.5)		6.9	2666.8	2659.9
SDNN	HIV–	45.5	43.2 (31.7–54.3)	<0.001	8.4	158.6	150.2
	HIV+	38.2	34.2 (24.7–47.7)		5.6	121.7	121.2
RMSSD	HIV–	49.2	43.3 (28.1–64.2)	<0.001	4.3	231.9	227.6
	HIV+	38.4	32.1 (20.3–47.4)		4.5	154.7	150.2
PNN50	HIV–	25.3	20.4 (3.6–41.0)	<0.001	0.0	73.3	73.3
	HIV+	15.2	6.9 (0.9–24.4)		0.0	76.0	76.0

IQR; inter quartile range, Min.; minimum, Max.; maximum, SDNN; standard deviation of the normal RR intervals, RMSSD; the root of the mean squares of successive RR differences, pNN50; the percentage of RR intervals greater than fifty milliseconds different from its predecessor, TF; total-frequency, LF; low-frequency and HF; high-frequency power.

The multivariable associations with all HRV outcomes are presented in Table 3. Model 2 includes age, sex, BMI, systolic blood pressure, smoking status, alcohol use, physical activity, total cholesterol and glucose. In addition to these variables, model 3 includes education and income. TG, HDL and LDL cholesterol were excluded due to multicollinearity with each other and/or total cholesterol. Diabetes was excluded due to multicollinearity with glucose. The multivariable models showed an inverse association between HIV treated with ART and log RMSSD and log pNN50.

**Table 3:** Association between HRV parameters and HIV.

Model	HIV		Model 1		Model 2		Model 3	

			Age and Sex		Model 1 + CVD risk factors		Model 2 + income & education	

	HIV		HIV		HIV		HIV	

	B Coef.	p-value	B Coef.	p-value	B Coef.	p-value	B Coef.	p-value

log SDNN	–0.19	<0.001	–0.10	0.05	–0.11	0.04	–0.11	0.057
log RMSSD	–0.27	<0.001	–0.15	0.05	–0.16	0.04	–0.16	0.04
log pNN50	–0.92	<0.001	–0.52	0.02	–0.59	0.01	–0.60	0.01
log TF power	–0.26	<0.01	–0.10	0.22	–0.11	0.20	–0.10	0.33
log LF power	–0.38	<0.001	–0.16	0.11	–0.14	0.18	–0.15	0.24
log HF power	–0.39	<0.01	–0.18	0.12	–0.20	0.09	–0.17	0.19

Model 2 includes BMI, physical activity, smoking status, alcohol use, systolic blood pressure, glucose and total cholesterol. HDL, LDL and TG were excluded due to multicollinearity with each other and/or total cholesterol. Diabetes was excluded due to multicollinearity with glucose. Coef.; coefficient, SDNN; standard deviation of the normal RR intervals, RMSSD; the root of the mean squares of successive RR differences, pNN50; the percentage of RR intervals greater than fifty milliseconds different from its predecessor, TF; total-frequency, LF; low-frequency and HF; high-frequency. Reference category: HIV-uninfected woman with low education, income < 600R, non-smoker, no alcohol use and physically active.

## 4. Discussion

HIV-infected participants treated with ART had a favourable CVD risk profile compared to HIV-uninfected participants, but showed significantly lower median values for all HRV parameters. HIV treated with ART was an independent risk factor for lower variability on log RMSSD and log pNN50, indicating a decreased functionality of the parasympathetic nervous system. HIV treated with ART was significantly associated with log SDNN, but this disappeared after additional adjustment for education and income. Overall, our findings indicate lower HRV for HIV-infected on ART. Since HRV has been shown to be an independent predictor of CVD, our findings of a lower HRV indicate a higher risk of CVD for HIV-infected treated with ART [8,9,10]. In addition, age was found to be associated with a decrease in all parameters of HRV and this translates to a decrease in the adaptation of the sympathetic and parasympathetic nervous system as one ages.

In contrast to most studies from high-income countries (HIC), we found a lower prevalence of conventional CVD risk factors in the HIV-infected population on ART compared to the HIV-uninfected population [4]. These findings are in line with other publications on CVD risk in SSA [24,25]. The difference in CVD risk profile between the HIV-infected population in HIC and low- to middle-income countries (LMIC) most likely reflects the differences in the HIV epidemic between HIC and LMIC. Whereas in HIC HIV infection is mainly seen in sub-populations like males having sex with males and injecting drug users, the epidemic in LMIC is affecting the general population. The lifestyle in HIC and LMIC also seem to differ, with a higher prevalence of smoking and obesity in the HIV-infected population in HIC whereas the HIV-infected populations in SSA are less often smokers and have a lower BMI compared to the non-infected population [4,25]. At current there is no gold standard CVD risk prediction tool for HIV-infected. The Data-collection on Adverse Effects of Anti-HIV Drugs (D:A:D) was developed specifically in HIV-infected populations and includes the use of ART [26]. However, this cohort included less than 25% females and only about 10% blacks. Recently several CVD scores, including the Framingham risk score and the D:A:D score, were compared in HIV-infected, and none of the scores turned out to be superior [27]. There is no research so far that compares CVD risk prediction tools in relation to HRV in HIV-infected.

The increased risk of CVD as indicated by a lower HRV in the HIV-infected population on ART that we observed is in line with a large body of literature suggesting that HIV infection increases the risk of CVD [4,5]. HRV has previously been shown to be related to CVD. In the Framingham Heart Study, a one–standard deviation decrement in log SDNN was associated with a hazard ratio of 1.47 for new cardiac events (95% confidence interval of 1.16 to 1.86) [28]. In the Atherosclerosis Risk in Communities Study, lifetime CVD risk was significantly increased for participants in the lowest compared to the highest tertile of the HRV outcomes LF/HF in men and SDDN, LF and LF/HF in women [29]. Our findings of a decreased HRV in the HIV-infected individuals treated with ART indicate a higher risk for CVD in the HIV-infected population in SSA, which needs to be confirmed by longitudinal studies assessing overt cardiovascular disease in SSA.

Limitations of this study are related to self-reported socio-demographic and lifestyle information which are potentially subject to social desirability and recall bias [30]. Furthermore, exposure to second-hand smoking has not been assessed even though this is a risk factor of CVD. Besides, blood pressure readings were taken with a wrist device, and not with a recommended arm-cuff device [31]. However, the same method was used for HIV-infected versus HIV-uninfected participants and therefore, this does not interfere with the comparison between both groups. A further limitation has been the use of five-minute HRV measurements. Although 24-hour HRV measurements are the golden standard, short five-minute HRV measurements have been considered methodologically adequate [32]. Finally, there was no information available on inflammatory markers, while this would have been valuable to gain insight into the pathway through which HIV/ART impact on HRV.

Major strengths of this study are the presence of an HIV-uninfected control group, allowing to gain insight into the role of HIV treated with ART on HRV and the taking into consideration of classic CVD risk for assessment of the effect of HIV treated with ART on CVD risk. As the study was undertaken in a general black rural sub-Saharan African population, the results may be generalizable to other rural SSA populations.

## 5. Conclusion

To conclude, we observed a favourable CVD risk profile based on traditional CVD risk factors in the HIV-infected cohort population treated with ART. However, HIV treated with ART was negatively associated with HRV, denoting a decreased functioning of the parasympathetic and sympathetic nervous system and thus a higher risk of CVD. Our findings suggest that HIV-infection treated with ART is an independent risk factor for CVD. This underlines the importance of optimizing detection and treatment of manageable CVD risk factors to reduce CVD risk in people with HIV on ART.

## Abbreviations

ART – Antiretroviral treatment

CVD – cardiovascular diseases

HRV – Heart rate variability

HIV – Human immunodeficiency virus

NCS – Ndlovu Cohort study

NMC – Ndlovu Medical Center

SDNN – Standard deviation of the normal RR intervals

RMSD – Root of the mean squares of successive RR differences

pNN50 – Percentage of RR intervals greater than fifty milliseconds different from its predecessor

LF – Low frequency
